# Adenosine Metabolism Pathway Alterations in Frontal Cortical Neurons in Schizophrenia

**DOI:** 10.3390/cells13191657

**Published:** 2024-10-06

**Authors:** Smita Sahay, Emily A. Devine, Christina F.-A. Vargas, Robert E. McCullumsmith, Sinead M. O’Donovan

**Affiliations:** 1 Department of Neurosciences & Psychiatry, University of Toledo College of Medicine and Life Sciences, Toledo, OH 43614, USA; smita.sahay@rockets.utoledo.edu (S.S.); sinead.odonovan@ul.ie (S.M.O.); 2 Department of Pharmacology and Systems Physiology, University of Cincinnati College of Medicine, Cincinnati, OH 45267, USA; 3 Neuroscience Institute, ProMedica, Toledo, OH 43606, USA; 4 Department of Biological Sciences, University of Limerick, Castletroy, Limerick V94 T9PX, Ireland

**Keywords:** adenosine kinase, equilibrative nucleoside transporters, ectonucleoside triphosphate diphosphohydrolases, ecto-5′-nucleotidases, neuromodulation, pyramidal neurons, anterior cingulate cortex, schizophrenia

## Abstract

Schizophrenia is a neuropsychiatric illness characterized by altered neurotransmission, in which adenosine, a modulator of glutamate and dopamine, plays a critical role that is relatively unexplored in the human brain. In the present study, postmortem human brain tissue from the anterior cingulate cortex (ACC) of individuals with schizophrenia (*n* = 20) and sex- and age-matched control subjects without psychiatric illness (*n* = 20) was obtained from the Bronx–Mount Sinai NIH Brain and Tissue Repository. Enriched populations of ACC pyramidal neurons were isolated using laser microdissection (LMD). The mRNA expression levels of six key adenosine pathway components—adenosine kinase (ADK), equilibrative nucleoside transporters 1 and 2 (ENT1 and ENT2), ectonucleoside triphosphate diphosphohydrolases 1 and 3 (ENTPD1 and ENTPD3), and ecto-5′-nucleotidase (NT5E)—were quantified using real-time PCR (qPCR) in neurons from these individuals. No significant mRNA expression differences were observed between the schizophrenia and control groups (*p* > 0.05). However, a significant sex difference was found in ADK mRNA expression, with higher levels in male compared with female subjects (Mann–Whitney U = 86; *p* < 0.05), a finding significantly driven by disease (t_(17)_ = 3.289; *p <* 0.05). Correlation analyses also demonstrated significant associations (*n* = 12) between the expression of several adenosine pathway components (*p* < 0.05). In our dementia severity analysis, ENTPD1 mRNA expression was significantly higher in males in the “mild” clinical dementia rating (CDR) bin compared with males in the “none” CDR bin (F_(2, 13)_ = 5.212; *p < 0.05*). Lastly, antipsychotic analysis revealed no significant impact on the expression of adenosine pathway components between medicated and non-medicated schizophrenia subjects (*p* > 0.05). The observed sex-specific variations and inter-component correlations highlight the value of investigating sex differences in disease and contribute to the molecular basis of schizophrenia’s pathology.

## 1. Introduction

Schizophrenia is a debilitating neuropsychiatric disorder that impacts over two million individuals in the United States alone [1]. This illness is characterized by a spectrum of symptoms that are categorized as positive, negative, and cognitive [2,3,4]. Positive symptoms are correlated with hyperactivity of the dopaminergic system and manifest as psychosis, hallucinations, and delusions among patients [5]. Negative symptoms, such as social withdrawal and flattened affect, and cognitive symptoms, such as impaired working memory and a slow information-processing speed, are often associated with hypofunction of the glutamatergic system [6]. The potent neuromodulatory purine adenosine monitors synaptic transmission, regulates the release of dopamine and glutamate from vesicles, and, ultimately, maintains neurotransmitter homeostasis via a system of receptors, enzymes, and transporters found in the brain in different cell types [7,8,9]. Since dopamine and glutamate disruption is central to the pathogenesis of schizophrenia [10], the precise role of the various components of the adenosine system is of specific interest in the investigation of this illness yet remains insufficiently explored.

Growing evidence supports the adenosine hypothesis of schizophrenia, which differentiates between intracellular and extracellular adenosine and states that decreased extracellular adenosine availability negatively impacts neuronal activity and overall brain metabolism and may be involved in the onset of schizophrenia [9,11]. One factor that may contribute to a hypoadenosinergic state is the increased expression of the chiefly astrocytic enzyme adenosine kinase (ADK), which functions to intracellularly phosphorylate adenosine to 5′-adenosine monophosphate (AMP). A transgenic mouse model of ADK overexpression resulted in an extracellular adenosine deficiency in the brain as well as cognitive and locomotor deficits that model schizophrenia-like endophenotypes [12]. Several studies support this finding, stating that a lack of extracellular adenosine disrupts the balance of dopamine and glutamate levels, which contributes to schizophrenia [11,13,14]. However, postmortem studies in the field do not report significant changes in ADK mRNA and protein levels. ADK mRNA expression was not significantly different between schizophrenia and control subjects in dorsolateral prefrontal cortex (DLPFC) tissue, a finding that extended to the cell-level investigation in astrocytes and pyramidal neurons in the same study [3]. Complementing this, another study reported no significant difference in ADK mRNA or protein levels in the anterior cingulate cortex (ACC) or DLPFC between schizophrenia and control subjects [15].

Several mechanisms exist to compensate for extracellular adenosine deficiency. Ectonucleoside triphosphate diphosphohydrolases (ENTPDs) catabolize extracellular adenosine triphosphate (ATP) to adenosine diphosphate (ADP) as well as ADP to AMP. AMP is then rapidly catabolized to adenosine via the rate-limiting enzyme ecto-5′-nucleotidase (NT5E) [13]. Adenosine synthesized in this manner exerts its influence by binding to the widespread inhibitory A_1_ adenosine receptor expressed in most brain regions, inducing tonic inhibition [16]. Deficits in either of the sequentially hydrolyzing enzymes ultimately impair extracellular adenosine availability. The same postmortem study that reported no significant differences in ADK mRNA expression also reported no significant differences in NT5E mRNA expression at the brain region or cell level, yet they reported significantly reduced ENTPD mRNA expression in DLPFC astrocytes in schizophrenia compared with control subjects, although this may have been an antipsychotic-mediated effect [3]. Similarly, ENTPD differential gene expression and enzyme activity levels were reduced in the striatum of patients with schizophrenia, while NT5E activity levels remained unchanged in the same study [17]. Reduced levels of ENTPD may have an impact on the availability of substrates for adenosine production, ultimately decreasing inhibitory A_1_ receptor activation. Adenosine binding to A_1_ receptors inhibits dopamine release via A_1_ and dopamine D_1_ receptor heterodimer formation; thus, loss of this inhibition results in the dopaminergic hyperactivity that is postulated to underlie the positive symptoms observed in schizophrenia [7,18,19].

In a preclinical study utilizing NT5E-knockout mice, the concept of NT5E-mediated formation of adenosine that specifically activates faciliatory A_2A_ receptors in the striatum was established [20]. These mice exhibited no locomotor sensitization to a sustained, low-dose (2.5 mg/kg) administration of amphetamines, suggesting a perseverance against hyperactive dopamine signaling. In the case of low ENTPD availability, which may lead to a hyperdopaminergic response, as suggested by postmortem findings, increased NT5E activity may provide a compensatory mechanism to sustain extracellular adenosine and activate A_2A_ receptors, promoting A_2A_ receptor and dopamine D_2_ receptor heterodimerization to combat hyperlocomotion [21]. However, postmortem studies indicate no significant difference in NT5E mRNA expression levels between schizophrenia and control subjects, perhaps indicating the lack of this compensatory mechanism against increased dopamine neurotransmission, exacerbating positive symptoms in the illness. Further studies on the availability and activity of the hydrolyzing ENTPD and NT5E enzymes are warranted to fully understand their function in the pathology of schizophrenia.

In addition to the extracellular adenosine produced via hydrolytic enzymes, a primarily glial cell process, adenosine may also be generated either from s-adenosyl homocysteine (SAH) or intracellularly following nucleotide degradation from AMP and released extracellularly via equilibrative nucleoside transporters (ENTs), a process primarily carried out in neurons [22,23]. Neuronally produced adenosine preferentially activates faciliatory A_2A_ receptors on neurons and glial cells, promoting neuroplasticity [24]. ENTs are bidirectional and equilibrate adenosine concentrations across plasma membranes [25]. In one study, ENT1 protein levels were reduced in elderly schizophrenia patients’ superior temporal gyri, but no change was observed in the ACC of the same patients [26]. Consistent with this finding, ENT1 mRNA levels were significantly decreased in DLPFC pyramidal neurons obtained from individuals with schizophrenia compared with non-psychiatrically ill control subjects [3]. Since ENTs are bidirectional, decreased levels of these transporters may either increase extracellular adenosine levels, leading to the activation of inhibitory A_1_ receptors, or decrease extracellular adenosine levels, leading to reduced A_1_ receptor-mediated tonic inhibition in the brain, as has been observed in ENT1-knockout mice [27]. ENT2, another equilibrative nucleoside transporter, also facilitates the transport of nucleosides across membranes but has a 2.8-fold lower affinity for adenosine and is relatively understudied in the pathogenesis of schizophrenia [28,29].

These findings support the idea of adenosine dysfunction in schizophrenia, yet not much is understood about the mRNA expression of adenosine pathway components in frontal cortical neurons in patients diagnosed with this illness. In the present study, we assayed the transcript expressions of ADK, ENT1, ENT2, ENTPD1, ENTPD3, and NT5E in an enriched population of pyramidal neurons in the postmortem ACC tissue of patients diagnosed with schizophrenia compared with non-psychiatrically ill sex- and age-matched patients. The ACC was selected since this is a brain region involved in emotional processing, cognition, and decision making and has been shown to be disrupted in schizophrenia [30]. We assayed changes in pyramidal neurons since genetic risk factors for schizophrenia converge on glutamatergic synapses, and pyramidal neurons primarily communicate with other neurons via glutamate [31]. We hypothesized that adenosine pathway component mRNA expression levels are altered in a manner that favors decreased extracellular adenosine levels in schizophrenia patients. Our aim was to provide an understanding of the neuromodulatory adenosine pathway in the brains of patients diagnosed with this challenging illness with the hope of encouraging future studies to develop novel adenosine-based therapeutic strategies.

## 2. Materials and Methods

### 2.1. Subjects

Human brain tissue was obtained postmortem from the Bronx–Mount Sinai NIH Brain and Tissue Repository. Proper consent was obtained from the next of kin of patients utilizing IRB-approved protocols. The samples included controls (individuals without a psychiatric illness diagnosis; *n* = 20) and patients diagnosed with schizophrenia (*n* = 20), sourced from the anterior cingulate cortex (ACC) brain region. The groups were matched for postmortem interval (PMI), pH, age, and sex (Table 1). After dissection, the brain tissue samples were immediately frozen and preserved at −80 °C for further experiments. Prior to death, diagnoses for all patients were confirmed individually by two psychiatrists through reviews of autopsy reports, medical records, and family interviews, following the Structured Clinical Interview for the Diagnostic and Statistical Manual of Mental Disorders, Fourth Edition (DSM-IV). The medication status was classified as “on” for subjects who had been taking antipsychotics within their last six weeks of life. The comprehensive demographic data are reported in Supplementary Table S1.

### 2.2. Laser Microdissection (LMD)

Enriched populations of ACC pyramidal cells were identified using Nissl staining techniques to conduct cell-level investigations of the mRNA expressions of ADK, ENT1, ENT2, ENTPD1, ENTPD3, and NT5E. The Leica Laser Microdissection 6 instrument (Leica Microsystems, Wood Dale, IL, USA) was used to accurately perform LMD and excise stained pyramidal neurons from the superficial (layers II–III) and deep (layers V–VI) grey matter of the ACC. The LMD procedures followed previously described established methods [3,32,33,34]. In brief, the frozen tissue sections were thawed at room temperature, rehydrated with distilled water, and Nissl-stained with an RNAse-free cresyl violet solution (1% cresyl violet and 1% glacial acetic acid; pH 4.0) (FD NeuroTechnologies, Columbia, MD, USA). Next, ethanol washes and histoclear treatment were performed, after which enriched pyramidal neuron populations (500 per subject) were identified by their morphology and excised from the ACC grey matter using LMD. Although these samples were abundantly enriched [33,34,35], they may have included neuropil, processes, or other small cells, such as interneurons. However, we have extensively published this method, exhibited successful neuron identification and enrichment (Supplementary Methods), and determined that LMD is an effective tool for capturing neurons from postmortem tissue [36]. In the present study, LMD was performed under a 40× objective lens with the following laser settings: a power of 24–25, an aperture of 4–5, and a speed of 8. The cells were collected into the caps of 0.5 mL tubes (Axygen, Union City, CA, USA) for each subject, incubated with 30 μL of PicoPure RNA extraction buffer (Applied Biosystems, Foster City, CA, USA) for 30 min at 42 °C, centrifuged for 2 min at 400× *g*, and stored at −80 °C until needed.

### 2.3. RNA Isolation, Reverse Transcription, and Complementary DNA (cDNA) Pre-Amplification

RNA was extracted from enriched pyramidal cell populations using the PicoPure RNA isolation kit according to the manufacturer’s instructions (Molecular Devices, Sunnyvale, CA, USA). The High-Capacity cDNA Reverse Transcription Kit (Applied Biosystems, Foster City, CA, USA) was subsequently utilized to synthesize cDNA using 10 μL of total RNA. TaqMan primers for ADK (ADK), ENT1 (SLC29A1), ENT2 (SLC29A2), ENTPD1 (ENTPD1), ENTPD3 (ENTPD3), and NT5E (NT5E), as well as for housekeeping genes cyclophilin A (PPIA), beta actin (ACTB), beta2-microglobulin (B2M), and glyceraldehyde-3-phosphate dehydrogenase (GAPDH), were utilized (Supplementary Table S2). For the pre-amplification polymerase chain reaction (PCR), the primers were pooled, diluted with RNase/DNase-free water to a final concentration of 0.2×, and mixed with cDNA and FastStart Universal Mastermix (Roche Life Sciences, Indianapolis, IN, USA). The PCR protocol included an initial denaturation step at 95 °C for 10 min, followed by 14 cycles of denaturation at 95 °C for 14 s, and annealing at 60 °C for 4 min. After pre-amplification, the samples were diluted 1:5 with RNase-free water and stored at −20 °C until use in the quantitative PCR (RT-qPCR) experiments.

### 2.4. Quantitative Real-Time Polymerase Chain Reaction (RT-qPCR)

qPCR reactions were performed in duplicate for each subject using 96-well optical reaction plates (Life Technologies, Carlsbad, CA, USA). The plates were analyzed using an Applied Biosystems detection system (ABI SteponePlus, Life Technologies, USA). Each reaction comprised a 20 μL mixture containing 3 μL of pre-amplified cDNA, 10 μL of mastermix, and a 1× concentration of each primer (Applied Biosystems, Life Technologies, USA). The reaction had an initial ramp time of 10 min at 95 °C, 40 cycles of amplification for 15 s at 95 °C, and 1 min at 60 °C for annealing. The negative controls included reactions without cDNA (non-template) and reactions without reverse transcriptase (no RT). The relative concentrations of the target transcripts (ADK, ENT1, ENT2, ENTPD1, ENTPD3, and NT5E) were calculated using a standard curve produced from the cDNA dilutions pooled from all subjects. The transcript levels were normalized to the geometric means of the four reference genes, which showed consistent expression across all subjects (Student’s *t*-test; *p* > 0.05).

### 2.5. Data Analysis

The qPCR datasets for each transcript were independently tested for a normal distribution using the D’Agostino and Pearson omnibus normality test and for the homogeneity of variance using the F-test. Since the relative concentration values were not normally distributed after being normalized to the standard curve generated from the pooled cDNA across datasets, the data were log-transformed. Spearman’s correlation analyses were conducted to assess the relationship between the different transcripts for all transcript pair combinations. Multiple regression analyses were conducted to examine the associations between dependent measures (transcript expression) and age and PMI. An analysis of covariance (ANCOVA) test was performed for significant associations.

If significant associations were not found, the data were analyzed using an unpaired two-tailed Student’s *t*-test, Welch’s *t*-test, or the Mann–Whitney test, depending on whether the criteria were met for normality and homogeneity of variance. The CDR scores were categorized by no dementia symptoms (bin 1), very mild to mild dementia symptoms (bin 2), and moderate to severe dementia symptoms (bin 3) and analyzed using the analysis of variance (ANOVA) test or the Kruskal–Wallis test depending on whether the criteria were met for normality and homogeneity of variance. To address multiple comparisons for all significant findings, Bonferroni’s post hoc analysis was performed.

An alpha level of 0.05 was used for the statistical tests. Outliers were detected with the ROUT method (Q = 0.1%). The data were analyzed using GraphPad Prism, version 10.2.3 (GraphPad Software, La Jolla, CA, USA), and Statistica, version 13.0 (Statsoft, Tulsa, OK, USA).

## 3. Results

### 3.1. mRNA Expression of Adenosine Pathway Components

**Figure 1:**
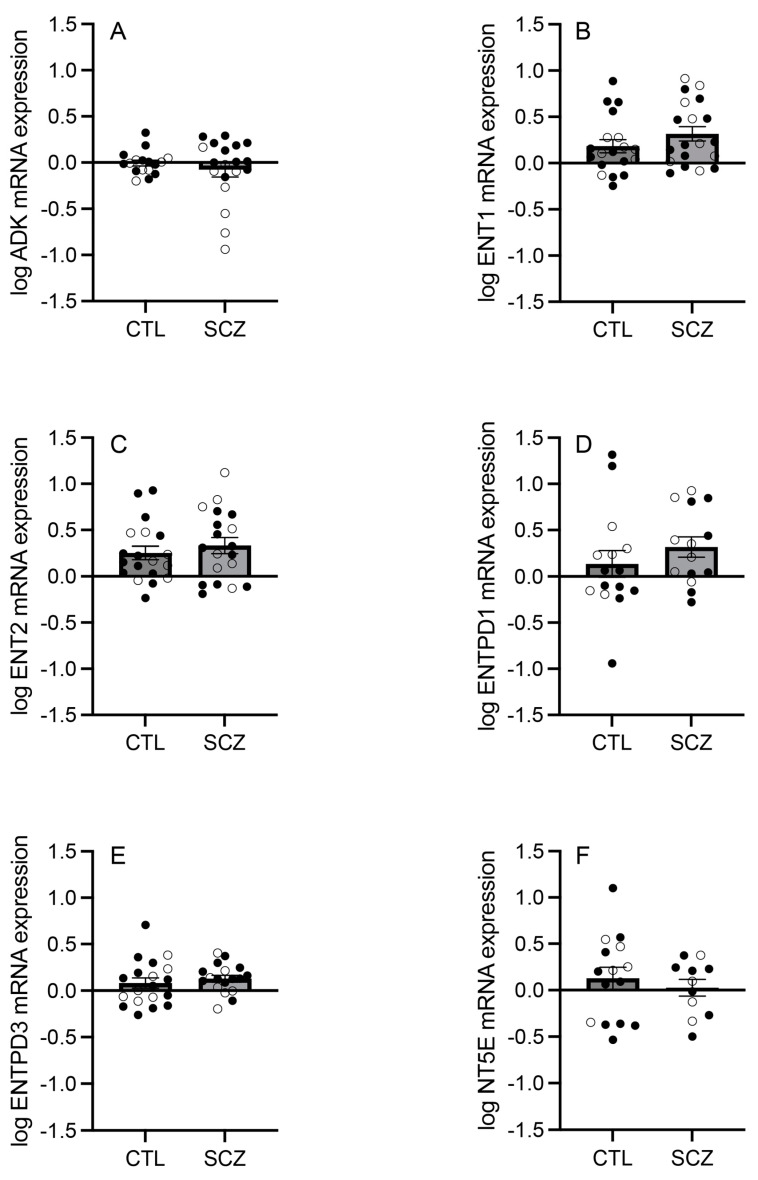
Expression of adenosine metabolism pathway components in an enriched population of anterior cingulate cortex (ACC) pyramidal neurons in control (CTL) vs. schizophrenia (SCZ) subjects. (**A**) ADK, (**B**) ENT1, (**C**) ENT2, (**D**) ENTPD1, (**E**) ENTPD3, and (**F**) NT5E mRNA expression levels were not significantly different between CTL and SCZ subjects. Open circles indicate females; closed circles indicate males. *n* = 11–19/group. Data are presented as means ± standard errors of the means (SEMs).

In an enriched population of ACC pyramidal neurons, the mRNA expression levels of ADK, ENT1, ENT2, ENTPD1, ENTPD3, and NT5E were assessed between individuals with schizophrenia and sex- and age-matched non-psychiatrically ill control subjects. ADK (Mann–Whitney U = 159; *p* = 0.950), ENT1 (t_(36)_ = 1.259; *p* = 0.216), ENT2 (t_(36)_ = 0.711; *p* = 0.482), ENTPD1 (t_(27)_ = 0.984; *p* = 0.334), ENTPD3 (t_(35)_ = 0.698; *p* = 0.490), and NT5E (t_(24)_ = 0.639; *p* = 0.529) mRNA expression levels were not significantly different between the two groups (Figure 1A–F).

### 3.2. mRNA Expression of Adenosine Pathway Components in Females versus Males

**Figure 2:**
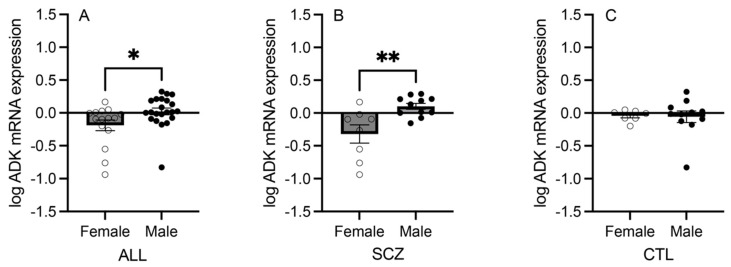
Expression of adenosine kinase (ADK) in an enriched population of anterior cingulate cortex (ACC) pyramidal neurons in female vs. male subjects. (**A**) ADK mRNA expression was significantly higher in male compared with female SCZ and CTL subjects combined. (**B**) ADK mRNA expression was significantly higher in male compared with female SCZ subjects only. (**C**) ADK mRNA expression was not significantly different between female and male CTL subjects only. Open circles indicate females; closed circles indicate males. *n* = 7–22/group. Data presented as means ± standard errors of the means (SEMs). * *p* < 0.05. ** *p* < 0.005.

In an enriched population of ACC pyramidal neurons, the mRNA expression levels of the adenosine metabolism pathway components were assessed between female and male schizophrenia and control subjects, between female and male schizophrenia subjects only, and between female and male control subjects only. Of the six transcripts, ADK mRNA expression was significantly higher in male compared with female schizophrenia and control subjects combined (Mann–Whitney U = 86; *p* = 0.014; Figure 2A). This change was driven by schizophrenia subjects, as the ADK mRNA expression level in the male schizophrenia group was significantly higher than in the female schizophrenia group (t_(17)_ = 3.289; *p* = 0.004; Figure 2B). The male and female control subject groups did not have significantly different ADK mRNA expression levels (Mann–Whitney U = 37; *p* = 0.930; Figure 2C). In the comparison including all schizophrenia and control subjects, the mRNA expression levels of ENT1, ENT2, ENTPD1, ENTPD3, and NT5E were not significantly different between the two sexes (Supplementary Figure S1).

### 3.3. Correlation Analyses: Intracellular Adenosine Metabolism and Adenosine Transport

**Figure 3:**
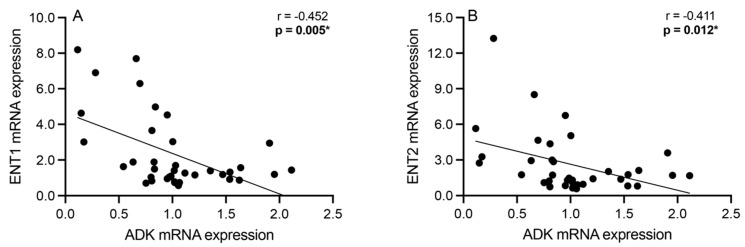
Spearman’s correlation analyses between adenosine kinase (ADK) and equilibrative nucleoside transporters 1 (ENT1) and 2 (ENT2). Significant negative associations were observed between (**A**) ADK and ENT1 mRNA expression and between (**B**) ADK and ENT2 mRNA expression across all schizophrenia and control subjects. *n* = 38/group. * *p* < 0.05.

Correlation analyses were performed to assess significant associations between the mRNA expression of the intracellular adenosine metabolism enzyme ADK and the bidirectional adenosine transporters ENT1 and ENT2 among all subjects. Significant negative associations were observed between ADK and ENT1 mRNA expression (Spearman’s r = −0.452; *p* = 0.005; Figure 3A) as well as between ADK and ENT2 mRNA expression (Spearman’s r = −0.411; *p* = 0.012; Figure 3B).

### 3.4. Correlation Analyses: Extracellular Adenosine Catabolism Enzymes

**Figure 4:**
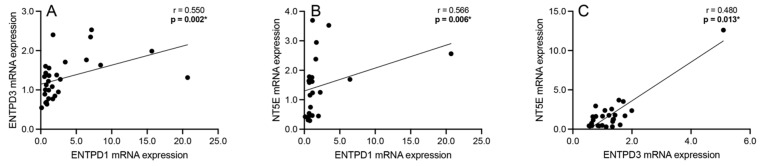
Spearman’s correlation analyses between ectonucleoside triphosphate diphosphohydrolase 1 (ENTPD1) and 3 (ENTPD3) and ecto-5′-nucleotidase (NT5E). Significant positive associations were observed between (**A**) ENTPD1 and ENTPD3 mRNA expression, between (**B**) ENTPD1 and NT5E mRNA expression, and between (**C**) ENTPD3 and NT5E across all schizophrenia and control subjects. *n* = 26–38/group. * *p* < 0.05.

Correlation analyses were performed to assess significant associations between the mRNA expression of the extracellular adenosine catabolism enzymes ENTPD1, ENTPD3, and NT5E among all subjects. A significant positive association was observed between ENTPD1 and ENTPD3 mRNA expression (Spearman’s r = 0.550; *p* = 0.002; Figure 4A), ENTPD1 and NT5E mRNA expression (Spearman’s r = 0.566; *p* = 0.006; Figure 4B), and ENTPD3 and NT5E mRNA expression (Spearman’s r = 0.480; *p* = 0.013; Figure 4C) across all subjects.

### 3.5. Correlation Analyses: Adenosine Transport and Primary Extracellular Catabolism Enzyme

**Figure 5:**
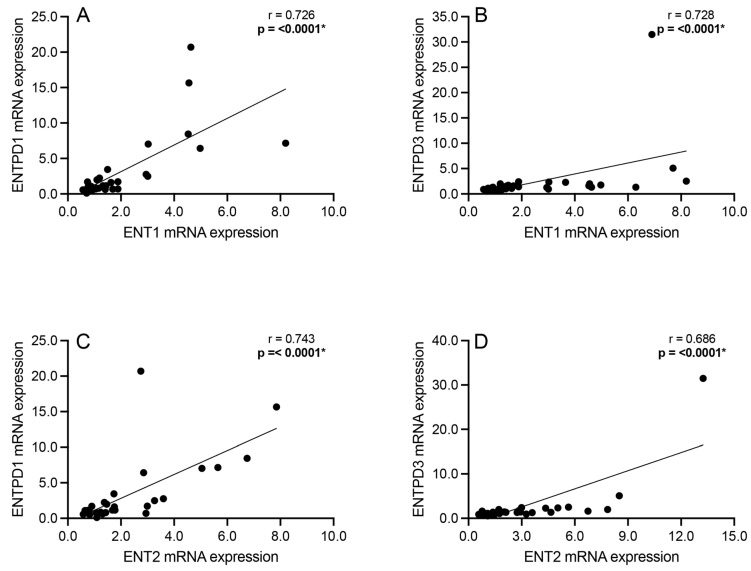
Spearman’s correlation analyses between equilibrative nucleoside transporters 1 and 2 (ENT1 and ENT2) and ectonucleoside triphosphate diphosphohydrolases 1 and 3 (ENTPD1 and ENTPD3). Significant positive associations were observed between (**A**) ENT1 and ENTPD1 mRNA expression, between (**B**) ENT1 and ENTPD3 mRNA expression, between (**C**) ENT2 and ENTPD1 mRNA expression, and between (**D**) ENT2 and ENTPD3 mRNA expression across all schizophrenia and control subjects. *n* = 29–38/group. * *p* < 0.05.

Correlation analyses were performed to assess significant associations between the mRNA expression of the bidirectional adenosine transporters ENT1 and ENT2 and the extracellular adenosine catabolism enzymes ENTPD1 and ENTPD3 among all subjects. Significant positive associations were observed between ENT1 and ENTPD1 mRNA expression (Spearman’s r = 0.726; *p* < 0.0001; Figure 5A), ENT1 and ENTPD3 mRNA expression (Spearman’s r = 0.728; *p* < 0.0001; Figure 5B), ENT2 and ENTPD1 mRNA expression (Spearman’s r = 0.743; *p* < 0.0001; Figure 5C), and ENT2 and ENTPD3 mRNA expression (Spearman’s r = 0.686; *p* < 0.0001; Figure 5D) across all subjects.

### 3.6. Correlation Analyses: Adenosine Transport and Rate-Limiting Extracellular Catabolism Enzyme

**Figure 6:**
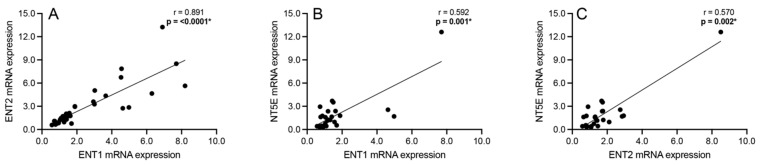
Spearman’s correlation analyses between equilibrative nucleoside transporters 1 (ENT1) and 2 (ENT2) and 5′-nucleotidase ecto (NT5E). Significant positive associations were observed between (**A**) ENT1 and ENT2 mRNA expression, between (**B**) ENT1 and NT5E mRNA expression, and between (**C**) ENT2 and NT5E across all schizophrenia and control subjects. *n* = 26–38/group. * *p* < 0.05.

Correlation analyses were performed to assess significant associations between the mRNA expression of the bidirectional adenosine transporters ENT1 and ENT2 and the rate-limiting component of the extracellular adenosine catabolism pathway, NT5E, among all subjects. Significant positive associations were observed between ENT1 and ENT2 mRNA expression (Spearman’s r = 0.891; *p* < 0.0001; Figure 6A), ENT1 and NT5E mRNA expression (Spearman’s r = 0.592; *p* = 0.001; Figure 6B), and ENT2 and NT5E mRNA expression (Spearman’s r = 0.570; *p* = 0.002; Figure 6C) across all schizophrenia and subjects.

### 3.7. mRNA Expression of Adenosine Pathway Components across CDR Bins

**Figure 7:**
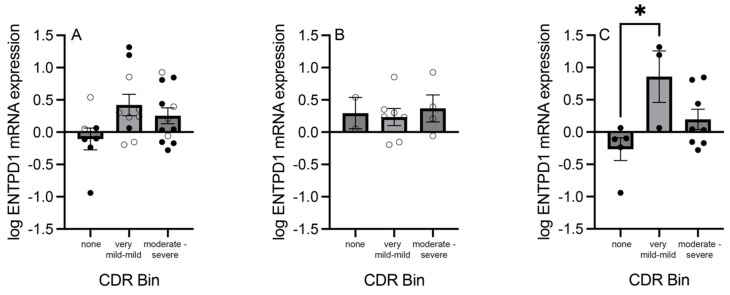
Expression of ectonucleoside triphosphate diphosphohydrolase 1 (ENTPD1) in an enriched population of anterior cingulate cortex (ACC) pyramidal neurons in control and schizophrenia subjects binned according to clinical dementia rating (CDR) scores. (**A**) ENTPD1 mRNA expression was not significantly different across CDR bins. (**B**) ENTPD1 mRNA expression was not significantly different across CDR bins in female subjects. (**C**) ENTPD1 mRNA expression was significantly higher in the “mild” CDR bin compared with the “none” CDR bin in male subjects (*F*_(2, 13)_ = 5.212; *p* = 0.022). Open circles indicate females; closed circles indicate males. *n* = 3–12/group. * *p* < 0.05.

In an enriched population of ACC pyramidal neurons, the mRNA expression of the six adenosine pathway components was analyzed based on CDR scores, binned into three groups: subjects with no dementia (bin 1; CDR score: 0), subjects with very mild to mild dementia (bin 2; CDR score: 0.5–1), and subjects with moderate to severe dementia (bin 3; CDR score: 2–3). When assessing mRNA expression by CDR bin and sex, ENTPD1 mRNA expression was significantly higher in male subjects in CDR bin 2 compared with CDR bin 1 (*F*_(2, 13)_ = 5.212; *p* = 0.022; Figure 7C), but ENTPD1 was not significantly different between CDR bins when assessing female subjects only (Kruskal–Wallis statistic = 0.332; *p* = 0.876; Figure 7B) or assessing female and male subjects combined (*F*_(2, 26)_ = 2.641; *p* = 0.090; Figure 7A). ADK, ENT1, ENT2, ENTPD1, ENTPD3, and NT5E mRNA expression levels were also not significantly different between all subjects combined across CDR bins (Supplementary Figure S2).

### 3.8. Antipsychotic Medication’s Effect on Adenosine Pathway

To assess whether medication influenced the mRNA expression of the adenosine pathway targets, an analysis was performed between individuals with schizophrenia who were taking antipsychotic medication at the time of death compared with individuals with schizophrenia who were not taking antipsychotic medication at the time of death. ADK, ENT1, ENT2, ENTPD1, ENTPD3, and NT5E mRNA expression levels were not significantly different between the two groups (Supplementary Figure S3).

## 4. Discussion

The present study explored the expression of core adenosine metabolism pathway components in an enriched population of pyramidal neurons from the ACC of individuals with schizophrenia and age- and sex-matched non-psychiatrically ill control subjects. The mRNA expression levels of ADK, ENT1, ENT2, ENTPD1, ENTPD3, and NT5E were not significantly different between the two groups (Figure 1), but sex differences and inter-component correlations were observed.

The mRNA expression of ADK was significantly greater in males than in females in schizophrenia and control subjects combined as well as in schizophrenia subjects alone, but not in control subjects alone (Figure 2), suggesting a disease effect. Higher levels of ADK may lead to increased intracellular adenosine clearance and greater ATP levels available to be released to the extracellular space [13]. Elevated ADK levels suggest enhanced intracellular substrate availability, but the lack of significantly elevated ENT1 mRNA levels in schizophrenia subjects could mean that adenosine transport may not be markedly altered. Notably, ADK is predominantly expressed in astrocytes [11], while this study focused on neurons, highlighting a mechanism that may not be exclusive to glial cells, as posited.

The observed sex difference supports the adenosine hypothesis of schizophrenia [11,12,13,14] and could help explain the fact that this illness is more prevalent among males [37,38]. Higher ADK levels may contribute to lower extracellular adenosine levels available to either dimerize with dopamine receptors or bind to adenosine receptors to modulate downstream dopamine and glutamate transmission [7,18,19]. Increased ATP levels may also lead to greater extracellular adenosine levels via catabolism, but significant increases in the mRNA expression of ENTPD or NT5E were not observed in schizophrenia subjects compared with controls (Figure 1) or in males compared with females (Supplementary Figure S1). This lack of a difference suggests that ATP catabolism may not be altered, ultimately resulting in a hypoadenosinergic state. We previously reported no significant differences in adenosine receptor mRNA expression in ACC pyramidal neurons between schizophrenia and control subjects [4]. Taken with the present findings, this may suggest a sex-specific intracellular adenosine metabolism perturbation rather than receptor-mediated pathology.

In addition, we observed several significant inter-component correlations between adenosine pathway enzymes and transporters. Significantly negative correlations were observed between ADK and ENT1 as well as between ADK and ENT2 (Figure 3). ENT1 and ENT2 are expressed at high levels in the brain, yet studies on ENT2 are in their early stages relative to ENT1 [39,40]. High levels of ENT1 and/or ENT2 on the neuronal cell surface suggest increased transport of adenosine across the cell membrane [14,41]. Given the negative correlation with low ADK levels, elevated ENT1 and/or ENT2 levels may be associated with greater extracellular adenosine levels. Adenosine, in the presence of increased extracellular adenosine deaminase (ADA) levels in neurons [3], is rapidly degraded to hypoxanthine, thereby depleting extracellular adenosine [42]. High levels of ADK in the context of lower levels of ENT1 and/or ENT2 imply a greater neuronal reliance on ATP, a substrate transported across the cell independent of ENT transporters, which, if not catabolized, may lead to a hypoadenosinergic extracellular state [43,44].

Significantly positive correlations were identified between components in the extracellular ATP catabolism pathway, specifically between ENTPD1 and ENTPD3, ENTPD1 and NT5E, and ENTPD3 and NT5E (Figure 4). ENTPD1 is primarily expressed in blood vessels [45] and is an important regulator of inflammation and immunity [46,47], while ENTPD3 is expressed at the highest level in the adult brain [48] and is, additionally, an important insulin modulator [49]. The positive correlations observed between the ENTPDs and NT5E suggest a coordinated regulation of ATP hydrolysis aimed at maintaining extracellular adenosine levels [50]. Since NT5E is also an important modulator of inflammation [46,51], a positive correlation with ENTPDs suggests a broader regulatory mechanism in response to neuroinflammation, which is implicated in the pathology of schizophrenia [52]. Thus, efficient and coordinated sequential ATP hydrolysis may represent a compensatory mechanism to maintain the homeostasis of the brain’s microenvironment during inflammatory episodes [53].

Significantly positive associations were also found between the ENTs and ENTPDs (Figure 5) as well as between the ENTs and NT5E (Figure 6). Elevated ENT levels in the presence of increased ENTPD and NT5E levels may imply a cyclical pattern of increased intracellular adenosine transport, leading to greater formation of ATP intracellularly, increased catabolism of ATP extracellularly, and, again, increased intracellular adenosine transport [23]. However, this study found a significantly negative correlation between the ENTs and ADK (Figure 3), suggesting greater adenosine transport extracellularly. Previous reports have found reduced ENT levels in different brain regions, such as the DLPFC [3] and superior temporal gyrus [26], in schizophrenia. Only one study investigated ENT1 in conjunction with NT5E and reported that ENT1-dependent release of adenosine was independent of NT5E activity in firing neurons [54], contrary to the present findings. To fully appreciate the physiological outcome of these mRNA expression correlations, imaging or protein-level confirmatory studies are necessary, especially given the bidirectional nature of ENTs [25].

An additional analysis was performed to assess the relationship between the adenosine pathway transcripts and CDR scores. The CDR is a tool that measures the impact of cognitive decline on daily functioning, with lower scores indicating little to no impact of cognitive decline on functioning and higher scores indicating the presence of dementia and greater functional disability [55]. Significant increases were not observed across the CDR bins for the transcripts among all subjects combined (Supplementary Figure S2); however, a significant increase was observed in ENTPD1 mRNA expression levels in males with very mild to mild dementia (bin 2) compared with males with no dementia (bin 1) (Figure 7). Investigations on the relationship between ENTPDs and dementia have reported mixed results. An increase in ENTPD2 levels was reported in the entorhinal cortex and precuneus postmortem tissue of subjects with Alzheimer’s disease at later stages of the disorder (stages III-IV and V-VI) compared with controls [56]. However, a decrease in ENTPD3 levels was found in the same comparisons, and sex differences were not investigated. A cerebral spinal fluid (CSF) proteo-genomic study investigating the leucine-rich repeat kinase 2 (LRRK2) protein implicated in the dementia severity seen among patients with Alzheimer’s and Parkinson’s disease found that LRKK2 exon missense variants were significantly associated with greater corresponding ENTPD1 CSF levels (*p* < 0.05) [57]. Our findings support the idea of ENTPD’s involvement in the pathogenesis of cognitive decline among patients with neuropsychiatric illnesses, specifically males; however, these findings must be further investigated to understand whether ENTPD modulation is an effective therapeutic strategy for cognitive decline.

Finally, to determine whether antipsychotic treatment impacted the mRNA expression of adenosine pathway components, a medication analysis was performed. No significant differences were observed for the adenosine targets between schizophrenia subjects on compared with off medication (Supplementary Figure S3). In a study measuring significantly altered adenosine targets in the DLPFC pyramidal neurons of rats treated with haloperidol for nine months, we reported a significant decrease in ENTPD2 mRNA expression in these rats compared with controls, but no differences were found in the same comparison for ENT1, ENTPD1, or NT5E [3]. Importantly, the present study had few subjects (*n* = 2–5/group) off medication, which is not alarming given the gravity of this illness. We also did not have access to information regarding other over-the-counter medications the patients may have been consuming prior to death. Thus, these findings need to be validated in larger studies that consider the effects of other medications, when possible.

Overall, our study investigated the transcriptional profile of select adenosine metabolic pathway components in ACC pyramidal neurons isolated from patients with schizophrenia to understand the molecular foundation of this illness. Although we observed significant sex differences and inter-component correlations at the mRNA level, future studies must assess protein levels and the activity of the adenosine components in larger studies utilizing tissues from other implicated brain regions to understand the functional pathological dynamics. Further imaging studies will also help visualize and quantify the functional and structural consequences of the molecular correlations observed in the brain. Given the inherent retrospective nature of postmortem studies, preclinical investigations are also warranted to test the postmortem findings observed. The results from these studies will collectively help investigators understand the potential of adenosine-based targets for therapeutic intervention in this challenging illness.

## 5. Conclusions

In conclusion, this study supports the adenosine hypothesis of schizophrenia and reports mRNA expression changes in adenosine metabolism pathway components in ACC pyramidal neurons isolated from individuals with schizophrenia compared with non-psychiatrically ill individuals. While no significant overall differences were observed between the schizophrenia and control groups for the six adenosine pathway enzymes and transporters of interest, notable sex differences and inter-component correlations emerged, suggesting complex regulatory mechanisms in the pathogenesis of this illness. The findings regarding dementia severity and medication effects should be validated in studies with larger cohorts. These findings, overall, contribute to our understanding of schizophrenia’s pathogenesis at an early molecular level and emphasize the need for broader research into the role of adenosine in this disorder.

## Figures and Tables

**Table 1:** Demographics of Mount Sinai NIH Brain and Tissue Repository subjects used in this study.

	N	Sex	Age	PMI (Hours)	pH	CDR	Medication
**Schizophrenia**	20	9F/11M	75 ± 8 (61–90)	13.1 ± 5.8 (5.8–24)	6.3 ± 0.2 (5.85–6.74)	2.3 ± 2 (0–3)	F: 4 on/3 off/2 Unk M: 8 on/3 off/0 Unk
**Control**	20	7F/13M	78 ± 7 (64–86)	12.4 ± 7.5 (3.3–24)	6.6 ± 0.4 (6.04–7.27)	0.4 ± 0.5 (0–3)	N/A

Data presented as means ± standard deviation. Data ranges are in parentheses. Abbreviations: N, number of subjects; F, female; M, male; PMI, postmortem interval; CDR, clinical dementia rating; Unk, unknown; N/A, not applicable.

## Data Availability

The data are contained within this Article and the Supplementary Materials.

## Supplementary Materials

The following supporting information can be downloaded at: https://www.mdpi.com/article/10.3390/cells13191657/s1. Supplementary Methods: Quality control studies demonstrating enrichment of pyramidal neurons in postmortem tissue; Table S1: Demographics of individual subjects; Table S2: TaqMan primers used in study; Figure S1: Expression of adenosine metabolism pathway components in an enriched population of anterior cingulate cortex (ACC) pyramidal neurons in female vs. male subjects; Figure S2: Expression of adenosine metabolism pathway components in an enriched population of anterior cingulate cortex (ACC) pyramidal neurons in control and schizophrenia subjects binned according to clinical dementia rating (CDR) scores; Figure S3: Expression of adenosine metabolism pathway components in an enriched population of anterior cingulate cortex (ACC) pyramidal neurons in schizophrenia subjects on vs. off antipsychotic medication; Supplementary References.
