# Supplementary material for: Adenosine Metabolism Pathway Alterations in Frontal Cortical Neurons in Schizophrenia
Source: Cells. 2024 Oct 6;13(19):1657. doi: 10.3390/cells13191657 (PMC11475131; doi:10.3390/cells13191657)
Supplement: Supplementary file 1 [file cells-13-01657-s001.zip › cells-3229646-supplementary.pdf]

Supplementary Materials.

Contents.

1. **Supplementary Methods** Page 2
2. **Supplementary Tables & Captions** Page 3
3. **Supplementary Figures & Captions** Page 5
4. **Supplementary References** Page 8

Supplementary Methods.

Quality control studies showing the enrichment of pyramidal neurons in postmortem brain tissue:

Laser microdissection (LMD) to isolate human postmortem tissue is a relatively new method. Previous publications have included detailed quality control studies for LMD-qPCR in human brain tissue [34], as shown in the supplemental materials of [3, 33]. These studies illustrate that the unique size and shape of pyramidal cells allow for their precise capture through Nissl staining and morphological identification.

In our process, we utilized postmortem tissue from the anterior cingulate cortex (ACC). Over approximately three hours, we consistently harvested 500 large cells with pyramidal-neuron morphology and 500 small cells from the same tissue section. Small cells, characterized by a circular, punctate nucleus, were presumed to be a mixture of glia and interneurons. These enriched cell populations were analyzed using qPCR, focusing on vesicular glutamate transporter 1 (VGLUT1) and neuron-specific enolase (NSE), which are markers for pyramidal neurons.

The large pyramidal neurons in the deep layer of the cortical matter were easily identifiable. The large cells showed enrichment for VGLUT1, while both large and small cells were equally enriched for NSE, indicating the presence of neurons in both populations. Overall, these quality control studies demonstrated that enriched populations of neurons can be harvested based on their morphological characteristics.

Supplementary Tables & Table Captions.

SUBJECT #	AGE	RACE	SEX	PH	PMI	CDR	MED STATUS	DIAGNOSIS
1	85	W	M	N/A	5.3	0	N/A	Control
2	78	W	M	N/A	8.1	2	N/A	Control
3	78	W	F	6.48	4.3	1	N/A	Control
4	75	B	M	6.43	5.0	0	N/A	Control
5	85	W	F	N/A	5.3	1	N/A	Control
6	86	W	F	N/A	11.5	0.5	N/A	Control
7	78	W	M	N/A	5.9	0	N/A	Control
8	85	N/A	M	N/A	16.2	0	N/A	Control
9	70	W	M	6.04	23.8	0	N/A	Control
10	64	W	M	6.12	10.4	0	N/A	Control
11	85	W	F	7.27	8.0	0	N/A	Control
12	68	W	F	6.30	24.0	0	N/A	Control
13	71	W	M	N/A	5.6	0	N/A	Control
14	75	H	F	N/A	3.3	0.5	N/A	Control
15	84	W	M	6.62	20.9	1	N/A	Control
16	71	W	M	7.09	21.7	0	N/A	Control
17	73	W	M	6.94	21.1	0.5	N/A	Control
18	86	W	F	N/A	10.2	0.5	N/A	Control
19	71	W	M	N/A	21.4	0.5	N/A	Control
20	84	H	M	N/A	16.8	0.5	N/A	Control
21	70	B	F	6.21	13.9	3	UNK	Schizophrenia
22	74	W	F	6.30	7.0	2	ON	Schizophrenia
23	81	W	F	5.93	12.5	0.5	OFF	Schizophrenia
24	82	W	F	5.89	8.8	1	OFF	Schizophrenia
25	86	W	F	5.80	18.2	3	ON	Schizophrenia
26	73	W	M	6.15	8.8	3	ON	Schizophrenia
27	90	W	F	5.97	7.8	0.5	ON	Schizophrenia
28	77	W	M	6.40	24.0	3	OFF	Schizophrenia
29	73	W	M	6.35	7.2	0	ON	Schizophrenia
30	70	W	M	6.49	14.3	1	ON	Schizophrenia
31	68	W	M	6.27	8.9	N/A	ON	Schizophrenia
32	86	W	M	6.48	15.4	3	ON	Schizophrenia
33	75	W	M	5.85	5.8	3	ON	Schizophrenia
34	81	W	F	6.47	15.1	3	OFF	Schizophrenia
35	70	W	M	6.36	17.3	3	OFF	Schizophrenia
36	75	W	F	6.49	21.5	0.5	UNK	Schizophrenia
37	62	W	F	6.74	23.7	0	ON	Schizophrenia
38	71	W	M	N/A	9.5	3	ON	Schizophrenia
39	83	W	M	N/A	16.3	3	OFF	Schizophrenia
40	61	W	M	N/A	6.2	2	ON	Schizophrenia

Supplementary Table 1. Demographics of anterior cingulate cortex (ACC) subjects from the Mount Sinai NIH Brain and Tissue Repository. Subject identification numbers, age, race, sex, pH, PMI, CDR, medication status, and diagnosis listed for all subjects used in study. *Abbreviations*: *PMI,* postmortem interval (hours); CDR, clinical dementia rating; MED, medication; *UNK*, unknown; *W*, white; *B*, black; *F,* female; *M* male; *N/A*, not available.

ASSAY	GENE NAME	PRIMER
Adenosine kinase	ADK	Hs00417073_m1
Equilibrative nucleoside transporter 1	SLC29A1	Hs01085706_m1
Equilibrative nucleoside transporter 2	SLC29A2	Hs01546959_g1
Ectonucleoside triphosphate diphosphohydrolase 1	ENTPD1	Hs00969556_m1
Ectonucleoside triphosphate diphosphohydrolase 3	ENTPD3	Hs00154325_m1
5'-nucleotidase ecto	NT5E	Hs00159686_m1
Cyclophilin A	PPIA	Hs99999904_m1
Beta Actin	ACTB	Hs99999903_m1
Beta2-Microglobulin	B2M	Hs99999907_m1
Glyceraldeyde-3-phosphate dehydrogenase	GAPDH	Hs99999905_m1

Supplementary Table 2. TaqMan primers. Assay, gene name, and IDs for all primers used in study.

Supplementary Figures & Figure Captions.

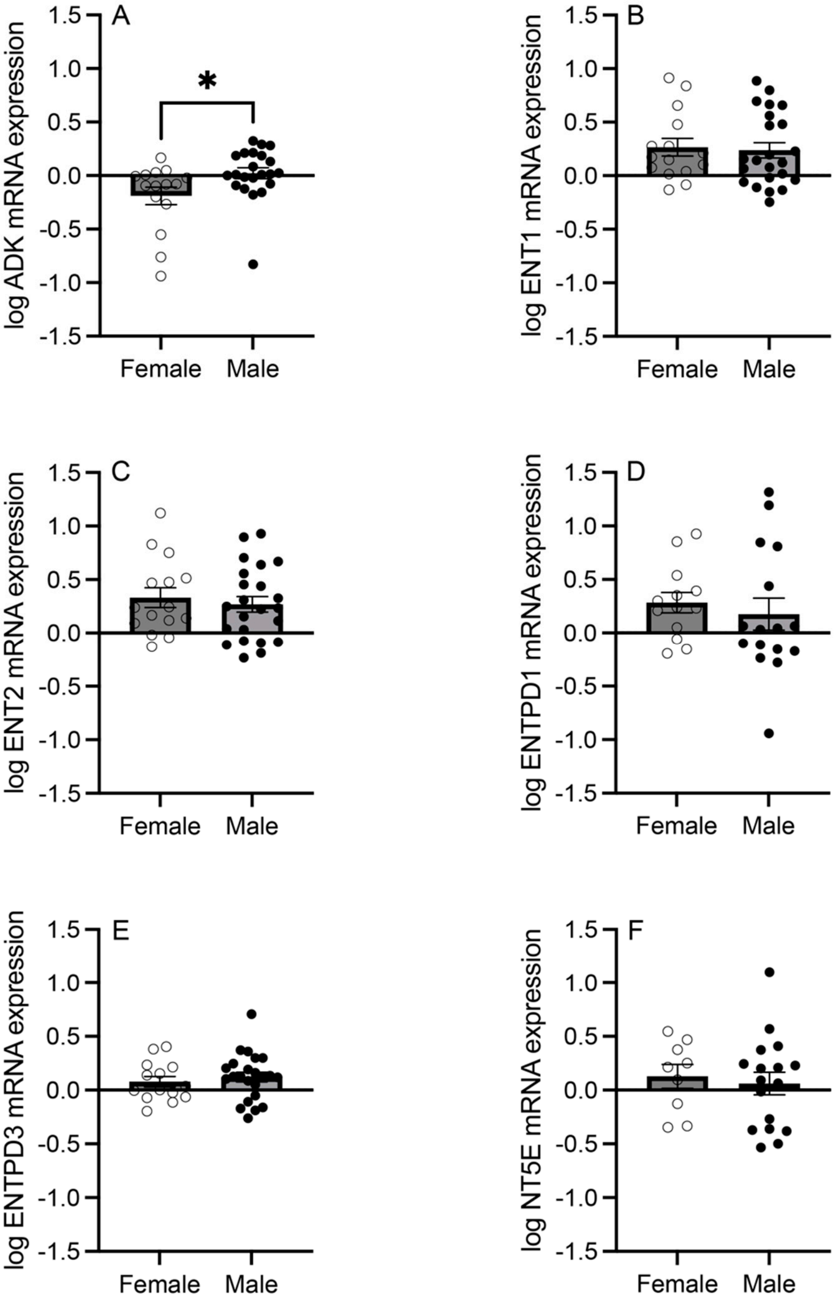

Supplementary Figure 1. Expression of adenosine metabolism pathway components in an enriched population of anterior cingulate cortex (ACC) pyramidal neurons in female vs. male subjects. (**A**) ADK mRNA expression was significantly higher in male compared to female subjects (*Mann-Whitney U* = 86, $p = 0.014$). (**B**) ENT1 ($t_{(36)} = 0.259$, $p = 0.797$), (**C**) ENT2 ($t_{(36)} = 0.539$, $p = 0.593$), (**D**) ENTPD1 ($t_{(27)} = 0.576$, $p = 0.569$), (**E**) ENTPD3 ($t_{(24)} = 0.340$, $p = 0.696$), and (**F**) NT5E ($t_{(35)} = 0.615$, $p = 0.543$) mRNA expression was not significantly different between female and male subjects. Open circles indicate females, closed circles indicate males. n = 9-23/group. Data presented as mean ± standard error of the mean (SEM).

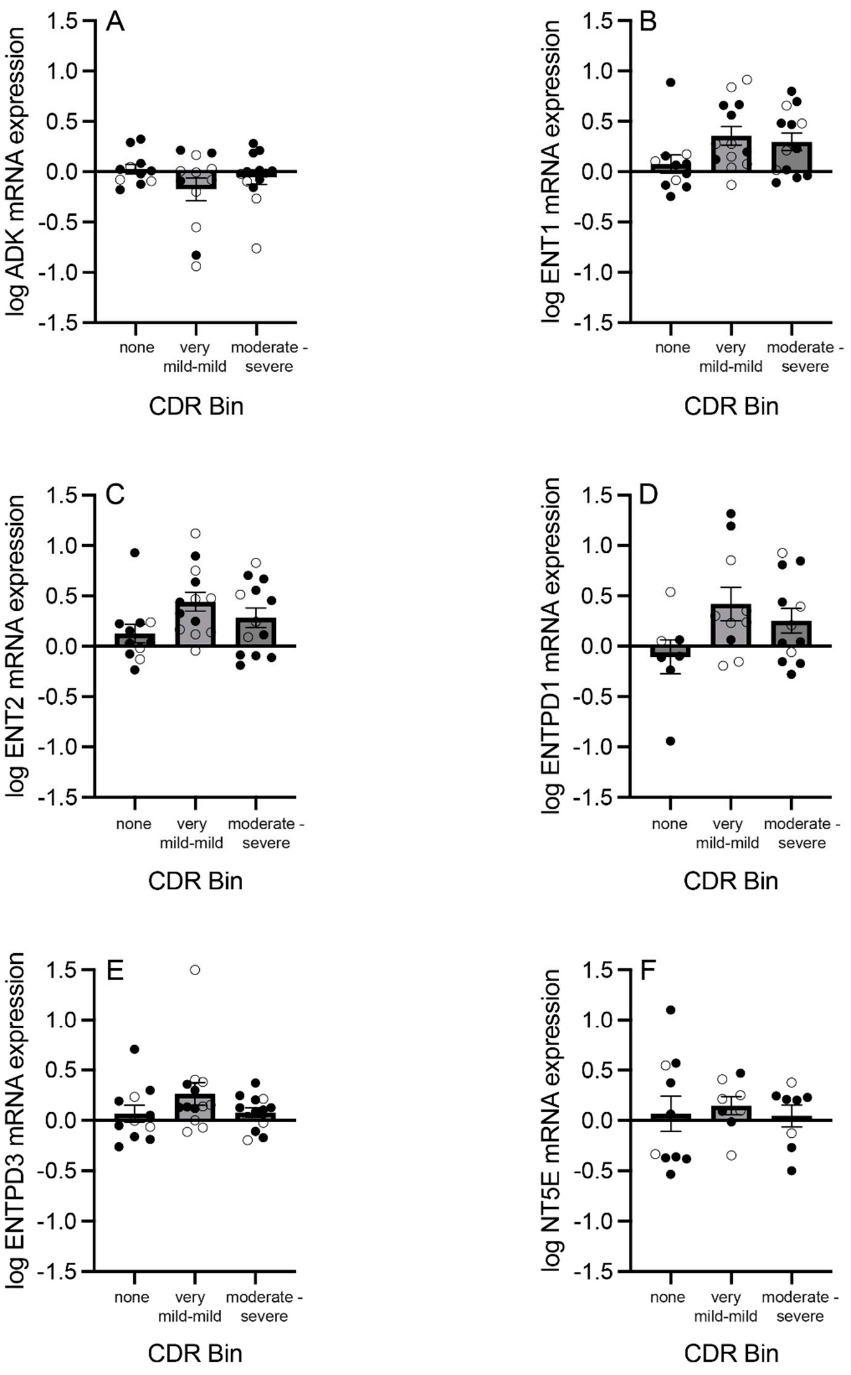

Supplementary Figure 2. Expression of adenosine metabolism pathway components in an enriched population of anterior cingulate cortex (ACC) pyramidal neurons in control and schizophrenia subjects binned according to clinical dementia rating (CDR) scores. (**A**) ADK (*Kruskal-Wallis statistic* = 1.143, p = 0.565), (**B**) ENT1 (*Kruskal-Wallis statistic* = 5.659, p = 0.059), (**C**) ENT2 (*Kruskal-Wallis statistic* = 5.406, p = 0.067), (**D**) ENTPD1 ($F_{(2,26)}$ = 0.233, p = 0.090), (**E**) ENTPD3 (*Kruskal-Wallis statistic* = 3.274, p = 0.195), and (**F**) NT5E ($F_{(2,23)}$ = 2.895, p = 0.137) mRNA expression was not significantly different among all subjects across CDR bins. Open circles indicate females, closed circles indicate males. n = 7-13/group. Data presented as mean ± standard error of the mean (SEM).

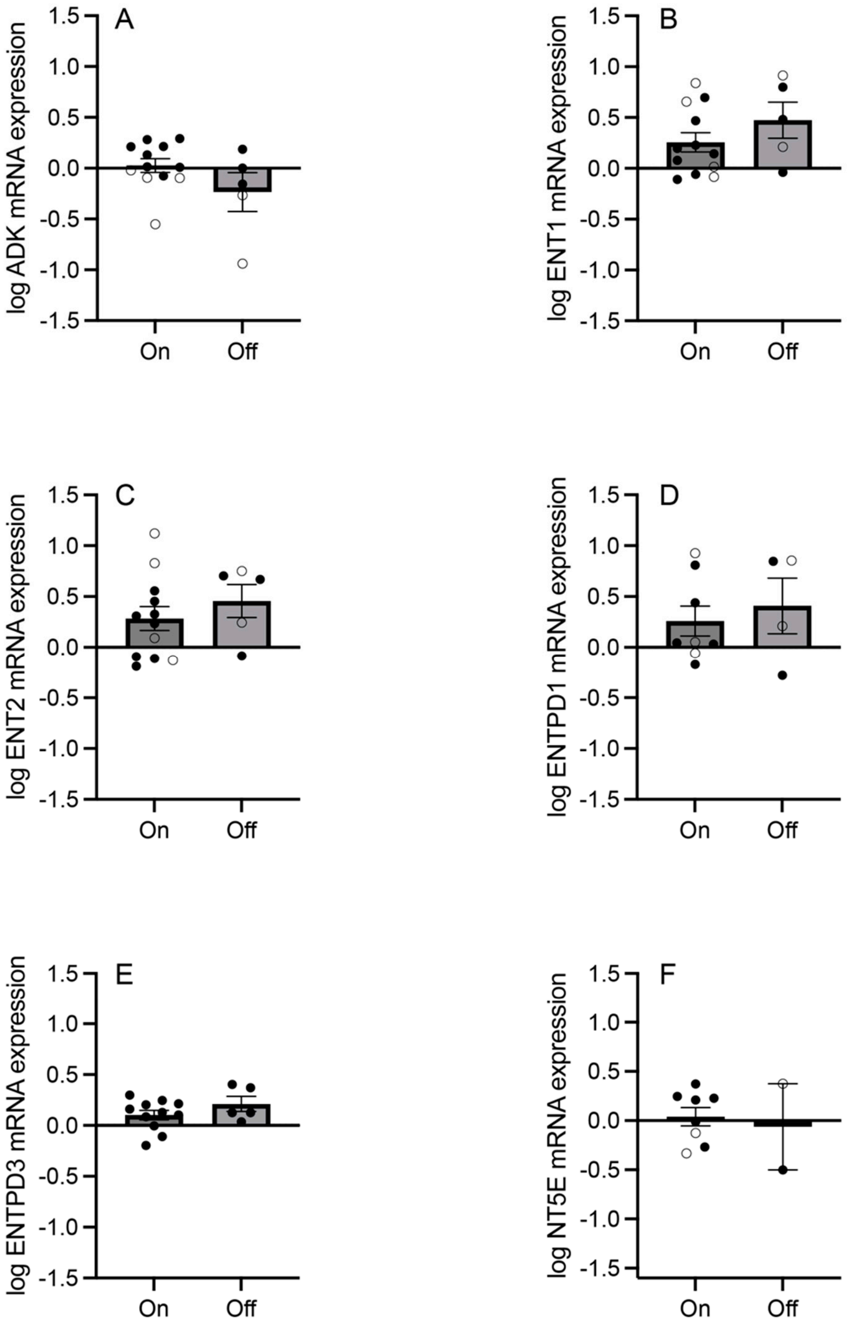

Supplementary Figure 3. Expression of adenosine metabolism pathway components in an enriched population of anterior cingulate cortex (ACC) pyramidal neurons in schizophrenia subjects on vs. off antipsychotic medication. (**A**) ADK ($t_{(15)}$ = 1.647, p = 0.120), (**B**) ENT1 ($F_{(1,14)}$ = 0.482, p = 0.499, after controlling for the effect of age), (**C**) ENT2 ($t_{(15)}$ = 0.819, p = 0.426), (**D**) ENTPD1 ($t_{(10)}$ = 0.529, p = 0.608), (**E**) ENTPD3 ($F_{(1,14)}$ = 0.505, p = 0.489, after controlling for the effect of age), and (**F**) NT5E (*Mann-Whitney U* = 9, p > 0.999) mRNA expression was not significantly different between schizophrenia subjects on medication compared to schizophrenia subjects off medication. Open circles indicate females, closed circles indicate males. n = 4-19/group. Data presented as mean ± standard error of the mean (SEM).

Supplementary References.

[3] S. M. O'Donovan *et al.*, "Cell-subtype-specific changes in adenosine pathways in schizophrenia," (in eng), *Neuropsychopharmacology,* vol. 43, no. 8, pp. 1667-1674, 07 2018, doi: 10.1038/s41386-018-0028-6.

[33] R. E. McCullumsmith *et al.*, "Cell-specific abnormalities of glutamate transporters in schizophrenia: sick astrocytes and compensating relay neurons?," (in eng), *Mol Psychiatry,* vol. 21, no. 6, pp. 823-30, Jun 2016, doi: 10.1038/mp.2015.148.

[34] M. S. Sodhi, M. Simmons, R. McCullumsmith, V. Haroutunian, and J. H. Meador-Woodruff, "Glutamatergic gene expression is specifically reduced in thalamocortical projecting relay neurons in schizophrenia," (in eng), *Biol Psychiatry,* vol. 70, no. 7, pp. 646-54, Oct 01 2011, doi: 10.1016/j.biopsych.2011.02.022.
